# Bittersweet Sugars: How Unusual Glycan Structures May Connect Epithelial-to-Mesenchymal Transition and Multidrug Resistance in Cancer

**DOI:** 10.3390/medicines10060036

**Published:** 2023-06-14

**Authors:** Leonardo Marques da Fonseca, Israel Diniz-Lima, Marcos André Rodrigues da Costa Santos, Tatiany Nunes Franklim, Kelli Monteiro da Costa, Ariely Costa dos Santos, Alexandre Morrot, Debora Decote-Ricardo, Raphael do Carmo Valente, Celio Geraldo Freire-de-Lima, Jhenifer Santos dos Reis, Leonardo Freire-de-Lima

**Affiliations:** 1 Instituto de Biofisica Carlos Chagas Filho, Universidade Federal do Rio de Janeiro, Rio de Janeiro 21941-902, Brazil; lfonseca@biof.ufrj.br (L.M.d.F.); israel@biof.ufrj.br (I.D.-L.); rodrigues8mr@gmail.com (M.A.R.d.C.S.); tnfranklim@gmail.com (T.N.F.); kellimc85@gmail.com (K.M.d.C.); arielycosta2101@gmail.com (A.C.d.S.); celio@biof.ufrj.br (C.G.F.-d.-L.); jhnffrrs8@gmail.com (J.S.d.R.); 2 Instituto Oswaldo Cruz, Fiocruz, Laboratório de Imunoparasitologia, Rio de Janeiro 21040-360, Brazil; alexandre.morrot@ioc.fiocruz.br; 3 Faculdade de Medicina, Universidade Federal do Rio de Janeiro, Rio de Janeiro 21941-902, Brazil; 4 Instituto de Veterinária, Departamento de Microbiologia e Imunologia Veterinária, Universidade Federal Rural do Rio de Janeiro, Seropédica 23890-000, Brazil; decotericardo96@gmail.com; 5 Núcleo Multidisciplinar de Pesquisa em Biologia, Universidade Federal do Rio de Janeiro, Campus Duque de Caxias, Rio de Janeiro 25250-470, Brazil; raphael.valente@caxias.ufrj.br

**Keywords:** glycoconjugates, cancer, glycosyltransferases, multidrug resistance phenotype, epithelial–mesenchymal transition process

## Abstract

Cancer cells are characterized by metabolic reprogramming, which enables their survival in of-ten inhospitable conditions. A very well-documented example that has gained attraction in re-cent years and is already considered a hallmark of transformed cells is the reprogramming of carbohydrate metabolism. Such a feature, in association with the differential expression of en-zymes involved in the biosynthesis of glycoconjugates, generically known as glycosyltransfer-ases, contributes to the expression of structurally atypical glycans when compared to those ex-pressed in healthy tissues. The latest studies have demonstrated that glycophenotypic alterations are capable of modulating multifactorial events essential for the development and/or progres-sion of the disease. Herein, we will address the importance of glycobiology in modern medi-cine, focusing on the ability of unusual/truncated *O*-linked glycans to modulate two complex and essential phenomena for cancer progression: the acquisition of the multidrug resistance (MDR) phenotype and the activation of molecular pathways associated with the epithelial–mesenchymal transition (EMT) process, an event deeply linked with cancer metastasis.

## 1. Unusual *O*-Linked Glycan Structures and Their Impact in Cancer Biology

Glycobiology is an exponentially growing field focused on the function, structure, evolution and biology of carbohydrates across all living organisms, being relevant to basic research, clinical medicine and biotechnology. On the other hand, the term glycomics refers to the studies that define the collection of glycans exhibited by a single cell or tissue given specific conditions, while glycoproteomics gives us the structure and location of glycan structures in the structure of a given protein [1,2]. Since the expression of glycoconjugates is a hallmark of all living cells, several research groups around the world are interested in understanding the biological role of different classes of glycomolecules, in both physiological and pathological conditions [3,4]. Over the last fifteen years, several important alterations in the expression of glycosyltransferases have been described. These changes lead to unusual glycosylation patterns for proteins within the cancer cell. Many of those alterations have proven to be highly effective as biomarkers, leading to useful diagnostic and prognostic predictions [5,6,7], including the ability to differentiate between benign and malignant [8]. Several instances of altered glycosylation patterns are either already being used or under study to be employed as biomarkers that can help in diagnoses or for predicting the prognosis of multiple diseases, including many types of cancer, diabetes and viral infections [9,10,11,12,13].

**Figure 1:**
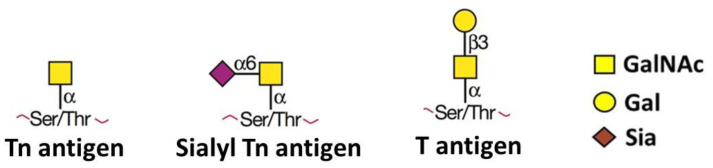
Tn, sialyl Tn and T antigens are the main examples of truncated *O*-linked glycans expressed in cancer cells. *O*-glycosylation is usually initiated in the Golgi apparatus, where C1GALT1, also known as T-synthase, adds galactose (Gal) from UDP-Gal to the common precursor Tn antigen (GalNAc*α*1-O-Ser/Thr) to generate T antigen (Gal*β*1-3GalNAc*α*1-O-Ser/Thr). Then, the T antigen can be modified by various glycosyltransferases to form many types of extended structures, which are usually found in many healthy tissues. In the absence of functional COSMC, which is necessary for the formation of functional T-synthase, or in the absence of functional C1GALT1 triggered by unknown mechanisms, Tn antigen may be used as a substrate by ST6GalNAc-I, which transfers a Sia unit (from CMP-Sia) to the Tn antigen to form STn. GalNAc—N-acetylgalactosamine; Gal—Galactose; Sia—Sialic Acid.

Thanks to biotechnological advances, the transformation of glycobiology from a descriptive and phenomenological discipline to one where the regulatory principles are not only understood, but effectively manipulated, has opened up new opportunities in the study of cancer and the search for effective therapeutic modalities [14,15,16]. An intriguing question that has attracted the attention of researchers around the world to the field of oncoglycobiology, is how structurally atypical glycans expressed by transformed cells are able to govern multiple events related to disease progression, such as the emergence of the multidrug resistance (MDR) phenotype [17], or the activation of the epithelial–mesenchymal transition process (EMT) [14], two of the most preeminent challenges faced by oncologists. Several tumor-associated carbohydrate antigens (TACAs), especially complex *N*-linked glycans, which are products of the glycosyltransferase *β*1,6-N-acetylglucosaminyltransferase V (GNT-V) activity, have been studied in many phenomena associated with development and/or cancer progression [18,19,20,21,22]. In this article, we will focus on a topic that still lacks its deserved attention in oncoglycobiology: the mechanisms involved on how *O*-linked glycans, which usually appear as truncated structures in transformed cells (Figure 1), may influence both the acquisition of chemoresistance, and the activation of signaling pathways linked to cancer metastasis. Examples of these structures include the T (Gal*β*1-3GalNAc*α*1-O-Ser/Thr), Tn (GalNAc*α*1-*O*-Ser/Thr) and Sialyl-Tn (STn) (NeuAc*α*2–6-GalNAc*α*1-*O*-Ser/Thr) antigens (Figure 1) [23]. We have known for almost 30 years that cancer patients exhibit changes in *O*-linked glycan expression patterns ever since Hakomori described such alterations in carcinoma-associated mucins [24]. Additionally, for over two decades, we have known that the expression of the STn antigen is related to a poor prognosis, regardless of tumor stage, grade or histological type [25]. The STn antigen has been described in most gastric carcinomas, as well as in other tumor tissues [26], and its expression is absent or uncommon in normal healthy tissues [26]. The molecular mechanisms underlying STn expression comprise the overexpression of the sialyltransferase ST6GalNAc1 [27] and/or the lack of C1GALT1 activity, also known as core 1 synthase. It compromises the function of the chaperone COSMC, essential for *O*-glycan chain elongation [28]. Although the association between truncated *O*-linked glycans and poor survival of cancer patients has been known for decades [23], the molecular mechanisms underlying such phenomena are still poorly understood. Since these truncated *O*-linked glycan structures are absent and/or expressed at low levels in healthy cells, many research groups have studied the possibility of developing glycovaccines with high therapeutic potential for different types of cancer [29,30].

In 2015, Hofmann and colleagues demonstrated that the knockdown of the COSMC chaperone, preventing *O*-glycan elongation beyond the initial GalNAc*α*1- residue on *O*-linked glycoproteins [31], was associated with elevated migration and reduced apoptosis of pancreatic cancer cells, suggesting that such a simple *O*-linked glycan may modulate the survival of transformed cells [31]. Recently, it has been revealed that the down-regulation of C1GALT1 in cholangiocarcinoma (CCA) promoted the expression of immature core 1 *O*-glycan, enhancing CCA progression, which was associated with the up-regulation of ABC transporter genes and anti-apoptotic proteins [32], which are both effects associated with the emergence of MDR phenotypes. In addition, C1GALT1 knockdown cells showed resistance to 5-fluorouracil via the activation of the AKT/ERK signaling pathway [32]. One of the glycoconjugates that are directly affected by overexpression of the STn antigen is MUC1. The elevated sialylation of MUC1 is capable of binding Siglec-9 and changing the tumor microenvironment, affecting cancer progression and changing macrophage polarization to a TAM-like phenotype, with increased PD-L1 expression [33]. A more recent study has shown that hypersialylated cancer cells increase siglec-7 and sigle-9 signaling, driving macrophage differentiation to an immunosuppressive phenotype, further supporting the notion that glycoconjugates can modulate immune checkpoints [34]. Additionally, a retrospective study on breast cancer patients showed that patients exhibiting a high STn/PD-L1 profile had poorer prognoses and might benefit from therapeutic strategies focusing on both fronts [35].

Over the past ten years, some works have demonstrated that the altered glycosylation in cancer cells seems to connect both EMT and MDR phenotypes [1,36,37]. In 2019, Thomas and colleagues proved that genetically deleting the COSMC chaperone in pancreatic ductal adenocarcinoma (PDAC) resulted in high expression of truncated *O*-glycans, which enhanced cell migration and invasion. The authors also observed high expression of mesenchymal markers in cells lacking the COSMC chaperone when compared to the parental cells [38]. These findings are in line with previous works showing that cancer patients positive for Tn and/or STn antigens exhibit poor prognoses [28,39,40]. In addition, it has been shown that these patients do not respond well to treatment with different chemotherapeutic agents, which supports the hypothesis of a correlation between the expression of truncated *O*-linked glycans and the acquisition of the MDR phenotype [25]. Similar results were obtained with T-synthase knock-out colorectal cancer cells. In this work, the authors observed that a deficiency of the glycosyltransferase enhanced oncogenic features via activation of the EMT process [41]. A paper from Pinho and coworkers in 2007 also showed that there is a correlation between increased STn expression, due to transfection of the glycosyltransferase ST6GalNAc-1/2, and activation of the EMT program. This forces the gastric carcinoma cells into a phenotype characterized by decreased cell-to-cell aggregation and augmented migration and invasiveness [42].

**Figure 2:**
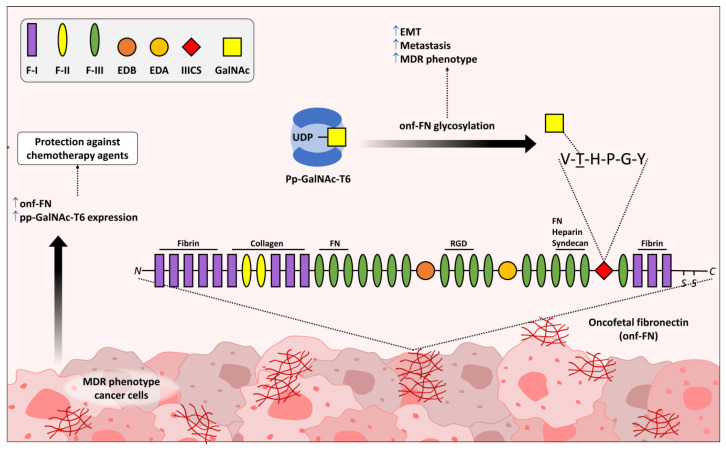
Representation of the simplified structure of fibronectin from the N-terminal to the C-terminal portion, with cysteine residues and their binding domains consisting of type I (F-1), II (F-II) and III (F-III), and repeat domains comprising the fibrin, collagen, arginylglycylaspartic acid (RGD), FN, heparin and syndecan binding domains. Between F-I and F-III, there is a type III connecting segment (IIICS), which is the major binding site for the integrin α4β1, also known as VLA-4. Different peptide motifs may also be detected in the FN structure, such as EDB and EDA. During alternative splicing, more than 20 fibronectin isoforms can be generated, among which, some carry the IIICS domain (also known as the variable region) that contains the hexapeptide VTHPGY, which is glycosylated at the Thr residue by the action of GALNT6, generating the *O*-glycosylated fibronectin. *O*-glycosylated fibronectin is able to modulate both the EMT process and the acquisition/maintenance of the MDR phenotype in cancer cells.

The non-sialylated version of the Tn antigen has also been shown to influence metastatic potential in colorectal cancer cells, due to an upregulation of the H-Ras proto-oncogene, a known activator of EMT. In the same study, transfecting the cells with COSMC and reducing Tn expression or knocking-down H-Ras stopped EMT activation [43,44]. In a recent review, Beaman and colleagues classified proteins from the UDP-N-α-D galactosamine:polypeptide N-acetylgalactosaminyltransferases family (GALNTs) as master regulators of metastatic processes, due to the ability of truncated *O*-glycans structures, such as Tn and STn antigens, to activate EMT [45]. Over the last ten years, our own research group has studied the expression and the biological effects induced by an atypical fibronectin (FN) isoform, named oncofetal FN (onf-FN) [19,31,46,47,48], which was initially described by Hakomori’s group in the 1980s in both cancer cells and embryonic tissues [49]. In the pioneer study, it was demonstrated that in the IIICS domain of FN (IIICS-FN), a unit of GalNAc can be transferred from UDP-GalNAc to the threonine (Thr) residue of the hexapeptide VTHPGY by the action of a polypeptide *N*-acetylgalactosaminyl transferase (GALNT6), generating the Tn antigen, which is the minimum saccharide epitope recognized by the FDC-6 mAb in the oncofetal glycoprotein [50]. Since its discovery, onf-FN continued to be used as a tumor biomarker for at least 25 years [51,52], and its procarcinogenic properties were described in 2011 in epithelial cells undergoing EMT [46]. Subsequently, further studies confirmed the initial findings [19,47,48]. More recently, the oncofetal glycoprotein was described in human macrophages (Mφ) exposed to anti-inflammatory signals [53], which show similar biological properties to tumor-associated Mφ (TAMs) [54], thereby modulating both the EMT process [55] and MDR phenotype [56]. Over the last twenty years, several papers have already described that extracellular matrix (ECM) components, including FN, protect cancer cells from cytotoxic insults induced by different chemotherapeutic agents [57,58,59,60]. Following that thread, we recently published a paper showing that inducing a MDR phenotype in breast cancer cells, leads to the overexpression of GALNT6. However, not only that, reducing the expression of GALNT6 also promoted a partial reversion of the resistance phenotype, showing that this glysosyltransferase, as well as onf-FN, may play a direct role not only in the activation of EMT and promotion of metastasis, but also in the acquisition of resistance phenotypes [61] (Figure 2). Other enzymes of the same family have been ascribed important roles in cancer progression. GALNT5, for instance, has been shown to promote an invasive phenotype in cholangiocarcinoma (CCA) cells that previously expressed it at low levels. As phosphorylation levels of ERK and Akt are increased in such cells when GALNT5 expression is increased, it is likely that its expression is associated with EMT activation [62]. GALNT14 overexpression in breast cancer MCF-7 cells was also shown to upregulate mesenchymal markers, such as N-cadherin and vimentin while downregulating epithelial markers, as is the case of E-cadherin, all the while stimulating cell migration and invasion capabilities [63]. Furthermore, GALNT14 is also capable of increasing drug resistance in MCF-7 cells. Its expression was found to be high in adriamycin-resistant cells, and its suppression sensitizes the cell to the drug. The effect is more directly due to an associated overexpression of P-gp, suggesting that glycoconjugates produced by GALNT14 are involved in the regulation of its expression [64].

## 2. Conclusions and Future Directions

Nowadays, both MDR phenotypes and the activation of EMT in transformed cells are the two most worrisome problems faced by both cancer patients and clinical oncologists. Over the last twenty years, the development of glycosyltransferase knockouts in mice have revealed that in vivo pathological phenotypes might be induced by the genetic manipulation of glycan structures, opening up a plethora of opportunities for studying the glycome to exploit the biological role of glycoconjugates in numerous cell types [65,66]. These groundbreaking findings reinforce the hypothesis that proteins decorated with unusual glycan structures may act as potential drug targets for chronic diseases. Innovative understandings into the functions and structure of glycomolecules present in all living cells could be useful to improve therapy, and may advance our capability to enhance the performance of therapeutic antibodies and potentiate the immune responses against different types of cancer. Additionally, works developed by many research groups attributing roles of unusual *O*-linked glycans and enzymes belonging to the GALNT family in both EMT and MDR phenotypes, helps cement our understanding that these phenomena cannot be separated as they are not only integral to cancer progression as a whole, but are indivisible, stemming from the same root. These exemplify the potential of the developing field of glycomedicine, which aims to target disease-related changes in carbohydrates. Together, a better comprehension of how atypical glycosylation patterns may influence tumor progression and malignancy may lead to a bright new day for clinical oncology.

## Data Availability

Not applicable.
